# Professionalism in Practice: A Novel Approach to Integrating Small Doses of Case-Based Professionalism Education Into Monthly Grand Rounds

**DOI:** 10.1177/23821205261449384

**Published:** 2026-05-07

**Authors:** Allison H. Taylor, Andrew Caruso, Kelley Arredondo, Ellen M. Friedman, Stacey R. Rose

**Affiliations:** 1 Department of Medicine, 3989 Baylor College of Medicine, Houston, Texas, USA; 2 Department of Medicine, Staff Hospitalist, Michael E. DeBakey VA Medical Center, 3989 Baylor College of Medicine, Houston, Texas, USA; 3 Department of Medicine, Houston VA HSR&D Center for Innovations in Quality, Effectiveness, and Safety, Michael E. DeBakey VA Medical Center, Improving Clinical Care, VA South Central Mental Illness Research, Education, Clinical Center, Center for Professionalism, 3989 Baylor College of Medicine, Houston, Texas, USA; 4 Department of Pediatric Otolaryngology, Center for Professionalism, 3989 Baylor College of Medicine; 5 Department of Medicine, Infectious Diseases Section and the Huffington Department of Education, Innovation and Technology, Center for Professionalism, 3989 Baylor College of Medicine, Houston, Texas, USA

**Keywords:** professionalism education, case-based teaching, collaborative problem-solving, professional competenci(es), internal medicine, grand rounds

## Abstract

**Introduction:**

Professionalism is a critical construct across the health professions, though learners and faculty report feeling underprepared to navigate difficult situations; education in professionalism is challenged by the absence of a universal definition, time constraints due to overcrowded curricula, and the lack of consistent messaging to learners and faculty.

**Methods:**

Professionalism in Practice (PiP) was developed to provide small doses (∼5 minutes) of case-based professionalism education as part of an existing educational forum (Grand Rounds) to optimize attendance of both learners and faculty. Each vignette highlighted a common professionalism challenge and was aligned with an AAMC professional competency. Sessions were facilitated by learners and faculty in the department, and introduced a consistent framework (i.e., pause, my perspective, other’s perspective, collaboration) for navigating difficult situations.

**Results:**

Post-session survey respondents (n=153 across 11 sessions) indicated that vignettes were relevant (93% [n = 142/153] agreed or strongly agreed), and that participants intended to change behavior (70% [n = 120/151] agreed or strongly agreed) and use the PiP framework in future practice (90% [n = 45/50] agreed or strongly agreed). Open-text comments revealed specific planned behavioral changes, such as pausing when frustrated or avoiding biased language in documentation.

**Conclusions:**

PiP addresses known challenges in professionalism education by adding case-based teaching to an existing educational forum attended by both learners and faculty, and by using a consistent definition of professionalism and framework for approaching common challenges. The initiative is adaptable to other institutions seeking to incorporate small doses of professionalism teaching as part of routine educational practices.

## Introduction

Professionalism is a core construct across medical training and practice,^1-4^ and lapses in professionalism as a trainee are associated with ongoing challenges later in one’s career.^5^ Despite its critical role, both learners and faculty report feeling underprepared to navigate difficult interpersonal or ethical situations while maintaining professional behavior.^6,7^ Compounding this challenge is the lack of consensus regarding the ideal modality of professionalism education.^8^ Barriers to implementing professionalism education include the absence of a universal definition of professionalism^9,10^ and time pressure or lack of room in health professions’ curriculum to add professionalism sessions.^11^ Institutional buy-in and ensuring professionalism education at all levels of training and practice have been recommended as strategies to promote positive professional behavior and address the hidden curriculum.^10,12-14^ Aligning professionalism education with a competency-based framework has also been purported as a strategy for improving professionalism education and assessment.^15^ Although originally developed for entering medical students, the American Association of Medical Colleges (AAMC) Premed Professional Competency Framework^1^ can guide the development of professionalism curricula across the educational continuum.^16^

The professionalism education literature describes a variety of instructional approaches, including self-study modules, small and large group discussions, workshops, and simulation exercises.^17-24^ These interventions have targeted individual groups such as medical students or residents; however, training both learners and faculty simultaneously may be critical to establish a shared understanding of professionalism expectations and foster a consistent, institution-wide approach to navigating professionalism challenges in educational and clinical settings. Another limitation of prior interventions is the focus on discrete topics, such as conflict resolution,^17,18^ which may inadequately address the range of professionalism challenges that may arise throughout the spectrum of one’s career. Moreover, there is a lack of data on participants’ intended behavioral change, making it difficult to assess programmatic impact beyond learner satisfaction.

In line with adult learning theory and given competing educational demands and limited curricular space, incorporating brief, repeated doses of professionalism education within existing educational forums^25,26^ may be a pragmatic and effective strategy to enhance retention and application of professional knowledge and skills. To further maximize retention and applicability of the content, it is important to use case-based teaching with realistic scenarios,^14,27^ and to align teachings with a competency-based framework, such as the AAMC Premed Professional Competency Framework,^1^ which provides a shared language for professional behavior and decision-making.

At our institution, the Center for Professionalism (the Center) is charged with creating a positive professional culture, through both education and remediation efforts. In partnership with the Department of Medicine (DOM), and to address the aforementioned gaps in professionalism education, the Center has created a longitudinal educational series, Professionalism in Practice (PiP), that incorporates competency-aligned professionalism case vignettes, monthly, as part of an existing educational forum: DOM Grand Rounds. While each vignette highlights a unique professionalism challenge, the sessions present a consistent approach to addressing the concern, namely to pause, consider multiple perspectives, and move towards a collaborative solution. In this descriptive study, we detail the development and implementation of this integrated professionalism education approach and report findings from participant evaluations, including perceived relevance, engagement, and intended behavior change.

## Methods

This study was a descriptive analysis of an educational intervention that incorporated both quantitative and qualitative data from voluntary post-session evaluations. The intervention took place within an auditorium in the Department of Medicine at Baylor College of Medicine in Houston, Texas and online via Teams. Evaluation data were collected from sessions delivered between January 2024 and April 2025.

### Curriculum Development

Leaders of the clinical curriculum within the DOM met with representatives from the Center to create a longitudinal professionalism educational intervention with the following learning objectives for participants: 1) recognize professionalism challenges that commonly arise in the clinical learning and working environment; 2) describe a framework for approaching professionalism challenges (pause, my perspective, other’s perspective, collaboration) and 3) identify strategies for behavior change to promote positive professionalism. The curriculum was designed as a series of case vignettes (Supplement A), each highlighting a common professionalism challenge and aligned with an AAMC professional competency.^1^ Vignettes were developed by a small group of faculty (organizing committee) with both medical and surgical specialty representation, with further refinement by session facilitators. The committee also created the PiP framework (Pause, My Perspective, Other’s Perspective, Collaboration) (Supplement B) to guide discussion following each case, with the goal of teaching participants to approach in-the-moment professionalism challenges using collaborative problem-solving strategies. Leaders of the PiP initiative obtained approval from the department Chair for the program to be integrated into an existing educational venue: DOM Grand Rounds.

### Setting, Preparation and Participants

Between January 2024 and April 2025, 11 PiP sessions were delivered approximately monthly, integrated as a 5-minute session at the beginning of DOM Grand Rounds. Participants included attendees of DOM Grand Rounds, primarily learners (medical students, interns, residents, fellows) and faculty. Participants had the option to scan a QR code or click a link to sign in for attendance. To meet criteria for inclusion in the study, participants needed to attend the session and complete the post-session survey; participants who attended the session but did not complete the post-session survey were not included.

Each PiP session was presented by a facilitator – an individual identified by the DOM leadership as demonstrating exemplary professionalism. Facilitators included faculty leaders (e.g., department chair, section chair, clerkship director, physician scientist) as well as selected learners (medical students, chief residents, and clinical fellows). Approximately two weeks before each session, the facilitator met with leaders of the PiP initiative for training and rehearsal. During the rehearsal, the facilitator was provided with a prepared, five-slide PowerPoint presentation, and had an opportunity to practice delivering the vignette and discussion, using the PiP framework. Facilitators were provided with feedback and tips (Supplement C) to ensure consistent presentation styles across sessions.

### Implementation of PiP Sessions at Grand Rounds

For each PiP session, the facilitator delivered a five-slide presentation, including 1) the institutional definition of professionalism, 2) the case vignette, 3) a suggested approach for navigating the case using the PiP framework, 4) a description of the relevant AAMC competency and 5) a QR code/link to a brief survey for participants to provide feedback. One team member from the Center was present at each session and the sessions were recorded (see Supplement D for a sample recording) for potential future use as an educational resource.

### Evaluation

Participants were invited to complete an anonymous, voluntary, post-session survey (designed and refined by all study authors). Using a QR code displayed during the session, participants rated their perceptions of the session using a 5-point Likert scale (*1 = Strongly Disagree, 5 = Strongly Agree*; Supplement E). Participants were asked the extent to which they agreed that: 1) the session addressed relevant professionalism competencies, 2) the session encouraged [them] to make changes in [their] behavior, and 3) you will use the framework (e.g., pause, consider multiple perspectives, find a collaborative solution) to promote professionalism in the future. Participants self-reported their institutional role (e.g., student, intern, resident, fellow, faculty, staff) and were asked to describe how they planned to change their behavior in the future using the framework, and what professionalism topics they would like to discuss in the future. The survey was separate from the evaluation used for overall grand rounds. Survey responses from the initial session (in January 2024) were reviewed by the study authors to ensure that the results reflected the desired outcomes.

Study data were collected and managed using Research Electronic Data Capture (REDCap) electronic data capture tools hosted at our institution, which is a secure, web-based software platform designed to support data capture for research studies.^28,29^ While the PiP initiative is ongoing, data collected from January 2024 through April 2025 were analyzed for this report.

### Statistical Analysis

Descriptive statistics were used to summarize participants’ responses to three post-session items assessing perceived relevance, behavioral change, and intended future use of the framework. Frequencies and percentages were calculated for each response option and an independent samples t-test was conducted comparing average attendance between PIP and non-PIP grand rounds. Qualitative data were coded by thematic analysis.^30^

## Ethics Approval and Consent to Participate

All methods were carried out in accordance with the Declaration of Helsinki and other relevant guidelines and regulations. Participation in survey completion was voluntary and all study data was anonymous. The protocol was reviewed and approved by the Baylor College of Medicine Institutional Review Board (protocol # H-54098) with a waiver of written informed consent.

## Results

### Characteristics of Survey Respondents

A total of 153 DOM Grand Rounds participants responded to the post-session survey. Institutional role data were available for 152 participants. Respondents represented a range of professional levels, including faculty (n = 60, 39%), students (n = 36, 24%), residents (n = 34, 22%), interns (n = 12, 8%), fellows (n = 6, 4%), staff (n = 2, 1%), and other roles (n = 2, 1%). Eleven PiP modules were presented between January 2024 through April 2025 with an average attendance of 101 (between 65 - 156 participants per session).

### Quantitative Results

**Figure 1.**
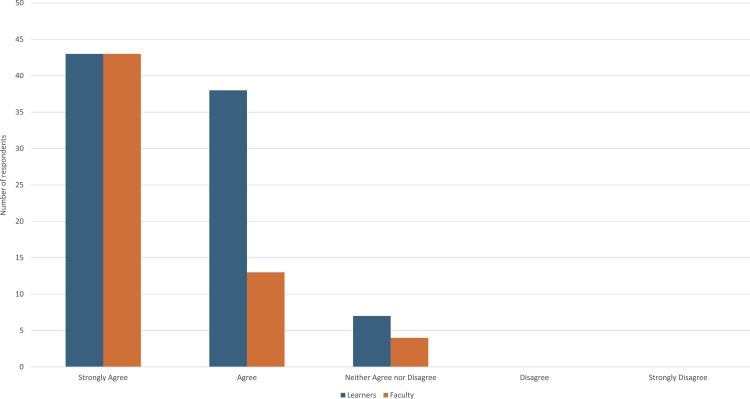
Extent the PiP session addressed relevant professionalism competency

As expected, average grand rounds attendance was similar between sessions that included PIP content (mean = 101) and those without PIP content (mean = 111), with no statistically significant difference observed (t(20) = 0.86, p = 0.40). The majority of respondents either strongly agreed (n = 90, 59%) or agreed (n = 52, 34%) that the session addressed relevant professionalism competencies (N = 153). Eleven participants (7%) neither agreed nor disagreed, and no respondents disagreed or strongly disagreed with the statement. Additionally, we compared responses across learners in relation to faculty responses on relevance of professionalism competencies (see Figure 1).

**Figure 2.**
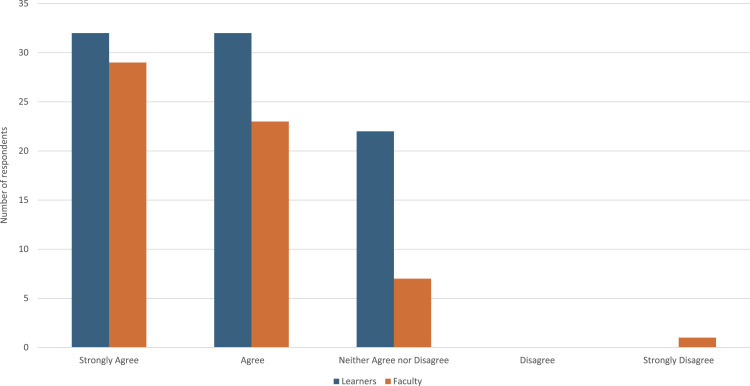
Extent the PiP session encouraged learners to change their behavior

When asked whether the session encouraged them to make changes in their behavior (N = 151), most participants responded positively, with 65 (43%) strongly agreeing and 55 (36%) agreeing. Thirty participants (20%) neither agreed nor disagreed, and one respondent (1%) strongly disagreed. In comparing responses for behavior change across learners and faculty, responses were similar across both groups (see Figure 2)

**Figure 3.**
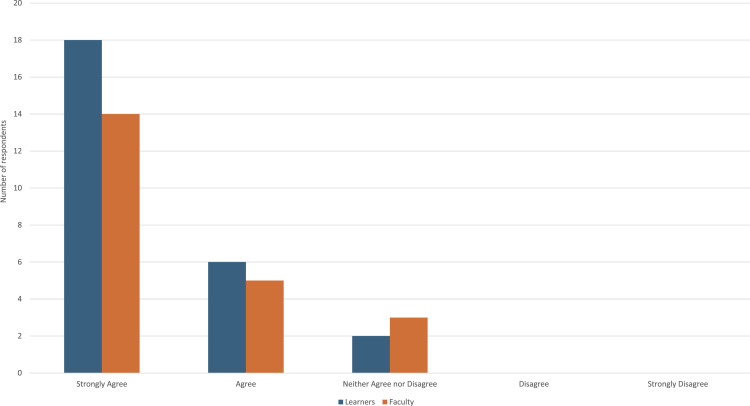
Extent learners agree they will use the PiP framework to promote professionalism in the future

Regarding the use of the PiP framework in future practice (N = 50), 34 respondents (68%) strongly agreed, 11 (22%) agreed, and 5 (10%) neither agreed nor disagreed. No participants disagreed or strongly disagreed.(Figure 2) In comparing responses regarding future use of framework across learners and faculty, responses were similar for both groups (see Figure 3).

### Qualitative Results

Twenty-four individuals provided free text responses on how they plan to change their behavior in the future using the PiP framework (See Table 1). Furthermore, nine individuals provided responses about the types of professionalism topics they would like to see discussed in the future. Respondents wanted sessions on conflict resolution (n = 2), bias or prejudice (n = 2), professionalism in documentation (n = 1), disclosure of errors (n = 1), and dealing with difficult patients (n = 1).

**Table 1.** Themes From Open Response on How Participants Will Change Future Behavior

Theme	Example quote	Frequency
Pause more before responding	“Take a pause when I notice I’m frustrated with a situation”	12
Reducing bias language in patient’s charts	“Thinking about what I put in the chart and trying to use less “loaded” words”	3
Giving or requesting specific feedback	“Great reminder to give specific feedback”	3
Acting with courtesy and respect	“Look at perspectives from all sides and act with courtesy and respect”	2

## Discussion

The Professionalism in Practice (PiP) initiative successfully incorporated small doses of case-based, professionalism education as part of regular, longitudinal programming within the DOM. Sessions were held at the beginning of Grand Rounds on a monthly basis, providing an opportunity for consistent messaging across learners and faculty, without requiring an additional educational session. Respondents found the cases to be practical and relevant to their experiences, and indicated they were likely to use the PiP framework in their future practice when navigating professionalism challenges. Additionally, several prior interventions cited in the literature have focused on a particular topic, such as conflict resolution,^17,18^ whereas our approach uses case vignettes to address a variety of relevant professionalism topics which may arise throughout the spectrum of one’s career. Our initiative is also innovative in soliciting feedback from participants on intended behavioral changes as part of programmatic assessment.

### Strengths and Limitations

Several elements have emerged as key to the impact of this initiative. First, buy-in from senior leadership was essential to allow PiP sessions to be incorporated into an already well-established educational conference (DOM Grand Rounds), and to help with the recruitment of facilitators. The creation of vignettes by an organizing committee, comprised of individuals with diverse clinical backgrounds and perspectives, was also helpful in designing cases that would resonate with a variety of learners and faculty. Aligning each vignette with an AAMC professional competency provided a roadmap for content creation and added educational credibility. Inviting DOM learners and faculty to serve as session facilitators fostered a sense of engagement across the department. The facilitator rehearsals were also critical to ensure that the sessions went smoothly and presented with consistent messaging of the PiP framework for managing professionalism challenges. Facilitators also helped to position the initiative as an educational opportunity rather than for punitive or remediation purposes.

PiP has been able to address known challenges in professionalism education; namely that it can be difficult to find time for learning about professionalism amidst the many other educational needs related to accreditation and continuing professional development, and that faculty and learners may receive mixed or differing messages about professional expectations.^13,14,24^

PiP was designed to address these challenges by incorporating small doses of professionalism education as part of an existing conference (DOM Grand Rounds) attended by both learners and faculty. Whereas other tools for teaching professionalism may be specific to one domain (such as conflict resolution^17,18^ or teamwork^31^), PiP teaches a simple, structured framework that can be used “in the moment” when experiencing a variety of professionalism challenges. This may explain why multiple participants – both in learner and faculty categories - were able to articulate specific plans for applying the framework in their future practice. The incorporation of PiP into a well-established educational series, attended by both learners and faculty, strategically offered consistent messaging across groups, further supporting a culture of professionalism across the department.

One potential concern when implementing professionalism education in a forum such as Grand Rounds is whether the content is perceived as relevant by senior faculty. The PiP curriculum was intentionally designed to address professionalism challenges encountered across the full career continuum, and facilitators included faculty leaders as well as learners. Survey findings demonstrated similarly high levels of perceived relevance and intended behavior change among faculty and learners, suggesting the content resonated broadly. Because PiP was delivered as a brief, five-minute segment integrated into existing Grand Rounds rather than as a standalone session, it did not replace traditional scientific content.

Furthermore, attendance at Grand Rounds was tracked by voluntary attendance registration with many participants opting to not sign in. Therefore, it is not appropriate to assess whether PiP influenced attendance. However, integrating professionalism education into an established educational venue may help ensure engagement across career stages without requiring additional time commitments. In fact, the average attendance count for the 11 Grand Round session with PiP modules was 101 and the average attendance for 11 Grand Rounds in 2025 that did not have PiP modules was 104. Because PiP was integrated into departmental Grand Rounds, it was not feasible to create a control group; thus, the findings are descriptive only. Future investigations are planned to compare professionalism knowledge and behaviors between departments that have implemented PiP and those that have not. Each PiP session was designed to be delivered in five minutes or less to facilitate its incorporation into the existing Grand Rounds structure; the downside of this approach is that there was limited time for participants to reflect on the case or interact with the facilitator. Nevertheless, the overall positive feedback from participants – including intended strategies for behavioral change – suggests that the “small dose” approach remained effective. Another limitation was that the development of each vignette was time intensive and required input from multiple faculty (organizing committee). The logistics of identifying facilitators and scheduling rehearsals were also substantial. Institutions seeking to replicate this educational initiative should be advised to plan ahead, identify committed faculty and a mechanism for administrative support to ensure an organized process. Cases were developed to resonate with an audience of learners and faculty within the DOM, which may limit their generalizability to other contexts. Still, since each case is aligned with an AAMC professional competency, it may be reasonable to make small adaptations to the vignettes as appropriate for other disciplines. Notably, at our institution, the success of the initiative within DOM has led to interest outside of the department, and now several medical and surgical departments have begun to incorporate PiP into their own departmental conferences; in most cases, only minor adaptations of the cases have been required.

The evaluation approach also had both strengths and limitations. While we intentionally kept the survey brief to facilitate completion in “real-time” (i.e. after the PiP session and before the “regular” grand rounds speaker), this limited the type and amount of data collected. From a process standpoint, we initially used the same QR code across all PiP sessions, which then required retrospective analysis to attribute the data to the correct date/vignette. Beginning in April 2025, we updated our processes such that each PiP session now has a dedicated QR code. The survey was entirely optional; with an average attendance of 101 (between 65 - 156 participants per session), a total of 153 DOM Grand Rounds participants responded to the survey. The consistency of positive feedback across a range of respondents (i.e., learners and faculty) enhances its validity. Given the novelty of the educational intervention, it was not possible to use an existing, validated survey tool, which may have impacted the accuracy of the findings. The evaluation approach also relied on self-report of acquired knowledge, skills or intended behavior change, consistent with levels 1 and 2 of the Kirkpatrick training evaluation model (i.e. reaction and learning); however, such learning does not always translate to behavior change.^32^ Hence, future initiatives should measure the observed impact of PiP upon professional behavior, such as though 360-degree evaluations or learning climate surveys.

An additional limitation is the lack of comparison data from other Grand Rounds presentations. Department of Medicine Grand Rounds encompasses a wide range of topics, including clinical advances, research findings, and specialty specific education, and do not routinely assess participants’ intended behavior change. Because PiP was specifically designed as a professionalism educational intervention, we included behavioral intention as an outcome to assess its potential impact. As a result, we are unable to compare these findings with responses to other Grand Rounds sessions. Future studies could examine behavioral outcomes across different types of educational programming to better understand the relative impact of professionalism focused interventions.

### Future Directions

PiP has become part of the fabric of DOM Grand Rounds at our institution, and we plan to continue developing new vignettes (each aligned with a AAMC professional competency) that will remain engaging to participants. Outside the DOM, faculty champions for PiP have emerged in a variety of medical and surgical fields, and we continue to refine our processes for launching the initiative in each new department. The program has also been adapted for interprofessional audiences. In October 2025, the PiP initiative was recognized with the inaugural Thomas J. Nasca Professionalism Award^33^ which will support future studies of the impact of PiP sessions on the professional behavior of learners and faculty, along with efforts to expand the initiative beyond our institution.

## Conclusions

PiP addresses known gaps in professionalism education by offering case-based teaching that is aligned with an established competency framework^1^ and models collaborative problem-solving to approach a range of professionalism challenges (the PiP framework). The integration of PiP at the beginning of Grand Rounds – an existing, well-attended educational venue – allows for longitudinal professionalism teaching without the burden of creating an additional course or conference. The curriculum is also novel in addressing professionalism education for multiple levels of learners; participants from a range of backgrounds found PiP cases to be relevant to their practice and reported intended behavioral changes. The program is easily adaptable and can facilitate the incorporation of small doses of professionalism education as part of routine departmental practices.

## Supplemental Material

Supplemental Material - Professionalism in Practice: A Novel Approach to Integrating Small Doses of Case-Based Professionalism Education Into Monthly Grand Rounds Supplemental Material for Professionalism in Practice: A Novel Approach to Integrating Small Doses of Case-Based Professionalism Education Into Monthly Grand Rounds by Allison H. Taylor, Andrew Caruso, Kelley Arredondo, Ellen M. Friedman and Stacey R. Rose in Journal of Medical Education and Curricular Development.

## Data Availability

The data that support the findings of this study are not publicly available due to institutional data privacy procedures. Data requests may be sent to the corresponding author for consideration pending institutional approval and agreement on the terms of data use.*
