# Supplementary material for: Professionalism in Practice: A Novel Approach to Integrating Small Doses of Case-Based Professionalism Education Into Monthly Grand Rounds
Source: J Med Educ Curric Dev. 2026 May 7;13:23821205261449384. doi: 10.1177/23821205261449384 (PMC13167374; doi:10.1177/23821205261449384)
Supplement: Supplemental Material - Professionalism in Practice: A Novel Approach to Integrating Small Doses of Case-Based Professionalism Education Into Monthly Grand Rounds [file sj-zip-1-mde-10.1177_23821205261449384.zip › A. Professionalism in Practice Vignettes.pdf]

AAMC Competency	Vignette Shorthand	Full Vignette
Commitment to Learning and Growth	Medical student receiving feedback	Christine, a third-year medical student, meets with Dr. Conn to receive feedback. Dr. Conn states: "You did not appear prepared for morning rounds, and your oral presentations seem disorganized and confusing."<br><br>Christine could respond,<br>"Well, I was up studying late at night and rounds start so early in the morning…I don't know what more I can do."<br>OR<br>"I am disappointed to hear this. Could you please provide some more details? What can I do better? Thank you."<br><br>Which would be the more helpful response, and why?
Commitment to Learning and Growth	Faculty giving feedback to resident	At the end of a 4-week rotation, Walter, a second year resident, meets with two faculty (Dr. Kim and Dr. Perez) to receive feedback.<br><br>Dr. Kim states: "You did a great job this month. Thanks!"<br><br>Dr. Perez states: "You were able to relay the daily clinical updates in a clear and logical progression, and were well prepared for rounds."<br><br>Which is the more helpful comment, and why?
Cultural Awareness & Humility	Medical documentation - patient leaving "AMA"	You are called to the ER to admit a patient who left the hospital "against medical advice" last week.<br><br>The discharge summary from that hospitalization mentions that the patient is "noncompliant," "refuses" lab draws and medication, and ends up leaving "AMA" despite significant risk of clinical decompensation.<br><br>You sigh and head down to see him, expecting confrontation.
Cultural Awareness & Humility	Patient making negative comment about a doctor's accent	You are the attending of an inpatient ward team comprised of a student, two interns and an upper-level resident.<br><br>During bedside rounds, a patient remarks that they want to be assigned a new doctor because "I can't understand anything this one says!" The patient points to indicate that he is referring to one of the interns, a Japanese female who moved to the United States for college.<br><br>How do you approach this situation?
Empathy and Compassion	Proud of grant; sharing accomplishments while being mindful of others' feelings	You just received notice that the competitive NIH grant you applied for three years in a row unsuccessfully was approved this year! You're excited and go to share the news with your colleagues.<br><br>In the course of telling a few colleagues one member looks at you and says 'That's not a big deal – I heard they were easier this year and were told to accept more'. You know that they were also recently applying for a similar kind of grant and did not get it.<br><br>You worked hard for this grant and are proud of this accomplishment so how do you respond?
Ethical Responsibility to Self and Others	Boundaries / social media	The resident social committee plans a party that is "BYOB." Over the course of the evening, several residents become intoxicated. A group photo is taken, and someone posts the picture to a public facing social media page with the caption, "Work hard, party harder!"<br><br>In clinic the following week, a patient comments on the posted photo: "Looks like you had a blast at that party! I bet you had quite a headache the next day!"

Interpersonal skills	Frustrated about bumped procedure	You are leaving the bedside of an angry patient whose procedure has been bumped AGAIN after being NPO all day.<br>Frustrated, you head back to your work room.<br>En route, you walk past the procedural specialist who appears to be heading home for the day.<br><br>What is a professional approach to this situation?
Oral Communication	Weekend call; consults called at 5 pm on a Friday	You are scheduled for weekend home call that starts at 5 pm Friday and ends 8 am Monday.<br><br>As soon as the clock strikes 5 pm, your phone buzzes with two consults from the hospitalist who has been taking admissions for the day.<br><br>You sigh in frustration, thinking, "why couldn't they have called these earlier?!"
Oral Communication	Using an interpreter, work life integration	You're in the midst of a very busy admitting day the Monday after a holiday weekend. The ER is busier than usual and you are called to admit three patients at once. You have practiced at Ben Taub for 10 years and feel fairly comfortable with speaking Spanish, but have no formal training and are not interpreter certified.<br><br>You are worried about your timing and promised your partner that you would be on time to dinner today so you weigh seeing the patient without the use of the interpreter service.
Resilience and Adaptability	Communicating with a frustrated patient re: imaging result	A patient sends three EPIC messages in a 24 hour period requesting a phone call to discuss an ultrasound report that was auto-released to the patient in the electronic medical record.<br><br>By the time you get home from the hospital and finish your clinic notes, it's 9:00 pm.<br><br>You call the patient and start to apologize for the late call, but she interrupts you, saying, "I can't believe you are calling me to discuss this at 9 o'clock at night, when I have been trying to reach your office all day! Have you no compassion? I am terrified about this result, and it seems like neither you nor anyone in your office gives a hoot!"
Service Orientation	Coverage for a colleague	You've just worked on a busy consult service for two straight weeks and while there were some very interesting cases while on service, you eagerly await your time off service. As you prepare to hand off the service to your colleague, they message back that something came up and they are unable to make it for the next two days. They don't give you any specific reason but politely ask if you can cover them.<br><br>How do you respond?
Team Work & Collaboration	Physician irritated about clinic - "Bea Grouchy"	Dr. Bea Grouchy comes to clinic, silently walks past the nurse's station, and enters the first clinic exam room without acknowledging the team.<br><br>In between patients, she complains to the nurses that there isn't enough equipment in rooms, the computer in the room is slow, and that there are too many no shows.
Team Work & Collaboration	Lack of respect for colleagues in another discipline ("why can't the surgeon manage the diabetes")	You are the upper level of a busy wards service. You receive a request from the emergency department for an admission: a 60 yo patient with well controlled diabetes presenting with a hip fracture. The orthopedics team has recommended medicine admission due to "medical complexity."<br><br>You turn to your team, roll your eyes, and say "This is so ridiculous. This is a surgical problem. Aren't the orthopedic surgeons DOCTORS?! They should be able to place basic insulin orders. Don't they know we're busy, too?!"<br><br>You look to the medical student for agreement – and then remember the student plans to apply for orthopedic residency.

Resilience and Adaptability	"Difficult" patient on the schedule	You are checking your clinic schedule for the week and notice one of your frequent, long-winded patients, Mr. S, is coming in for a follow up visit.<br><br>You have previously had trouble solving some of this patient’s numerous complaints and dread when this patient comes in.<br><br>As the day arrives, you comment to the clinic staff: “here comes Mr. S again…he always makes me run late with his litany of complaints. I need to figure out how to get this patient out of my panel!”
