# Supplementary material for: Professionalism in Practice: A Novel Approach to Integrating Small Doses of Case-Based Professionalism Education Into Monthly Grand Rounds
Source: J Med Educ Curric Dev. 2026 May 7;13:23821205261449384. doi: 10.1177/23821205261449384 (PMC13167374; doi:10.1177/23821205261449384)
Supplement: Supplemental Material - Professionalism in Practice: A Novel Approach to Integrating Small Doses of Case-Based Professionalism Education Into Monthly Grand Rounds [file sj-zip-1-mde-10.1177_23821205261449384.zip › C. Tips for PiP Facilitators.pdf]

Baylor College of Medicine CENTER FOR PROFESSIONALISM

Professionalism in Practice (PiP) – Facilitator Guide

Tips for a successful session:

- Rehearse with Center for Professionalism leadership ~2 weeks prior to the PiP session to ensure familiarity with the case and discussion.
- Open the presentation by restating the goals of PiP (i.e. to ensure shared understanding of professionalism and to enhance skills in responding to commonly encountered challenges (not in response to a problem with professionalism across the department).
- Remind participants of the BCM definition of professionalism.
    - "A set of attitudes and behaviors that cultivates competence and connection through positive, respectful and trustworthy relationships."
- After presenting the case, emphasize that regardless of the situation, the PiP framework can be applied.
    - Pause, consider your perspective, the other's perspective, and then develop a collaborative solution.
- Pause for emphasis of the role of pausing to collect one's thoughts, then proceed with presenting the suggested approach to the case.
    - See notes in the slide deck.
- Keep the presentation to 5 min out of respect for the Grand Rounds speaker
    - Note, there will not be time for participant interaction.
- Show QR code for post session feedback survey and copy/paste link into the chat (for virtual participants).
- Work with faculty champion to share session recording (if available) with Center for Professionalism.
