# Supplementary material for: Professionalism in Practice: A Novel Approach to Integrating Small Doses of Case-Based Professionalism Education Into Monthly Grand Rounds
Source: J Med Educ Curric Dev. 2026 May 7;13:23821205261449384. doi: 10.1177/23821205261449384 (PMC13167374; doi:10.1177/23821205261449384)
Supplement: Supplemental Material - Professionalism in Practice: A Novel Approach to Integrating Small Doses of Case-Based Professionalism Education Into Monthly Grand Rounds [file sj-zip-1-mde-10.1177_23821205261449384.zip › E. PiP Evaluation.pdf]

Professionalism in Practice Post Survey

Thank you for attending today's session. Please fill out the following survey to help us improve our sessions.

Please read the consent form here.

[Attachment: "PiP consent.doc"]

By selecting yes, you voluntarily consent to complete this anonymous questionnaire evaluating the effect of a brief educational intervention on professionalism behavior.

- ◯ Yes
- ◯ No

To what extent do you agree today's session addressed relevant professionalism competencies?

- ◯ Strongly Agree
- ◯ Agree
- ◯ Neither Agree or Disagree
- ◯ Disagree
- ◯ Strongly Disagree

To what extent do you agree today's session encouraged you to make changes in your behavior?

- ◯ Strongly Agree
- ◯ Agree
- ◯ Neither Agree or Disagree
- ◯ Disagree
- ◯ Strongly Disagree

To what extent do you agree you will use the framework (e.g., pause, consider multiple perspectives, find a collaborative solution) to promote professionalism in the future?

- ◯ Strongly Agree
- ◯ Agree
- ◯ Neither Agree or Disagree
- ◯ Disagree
- ◯ Strongly Disagree

Briefly describe one strategy you plan to apply in your future practice to promote professionalism.

____

Which title best describes your current role?

- ◯ Student
- ◯ Intern
- ◯ Resident
- ◯ Fellow
- ◯ Faculty
- ◯ Staff
- ◯ Other

Current role

____

Which best describes your fellow title?

- ◯ Clinical Fellow
- ◯ PhD Fellow

What is your highest level of training?

- ☐ DO
- ☐ PA
- ☐ PhD
- ☐ MD
- ☐ MD/PhD
- ☐ NP
- ☐ Other

Highest level of training

____

Which BCM school are you affiliated with?	◯ Graduate School of Biomedical Sciences<br>◯ National School of Tropical Medicine<br>◯ School of Health Professions<br>◯ School of Medicine<br>◯ Other
Which school are you affiliated with?	__________________________________
What topics would you like to see in future professionalism presentations?	__________________________________
