# Supplementary material for: Professionalism in Practice: A Novel Approach to Integrating Small Doses of Case-Based Professionalism Education Into Monthly Grand Rounds
Source: J Med Educ Curric Dev. 2026 May 7;13:23821205261449384. doi: 10.1177/23821205261449384 (PMC13167374; doi:10.1177/23821205261449384)
Supplement: Supplemental Material - Professionalism in Practice: A Novel Approach to Integrating Small Doses of Case-Based Professionalism Education Into Monthly Grand Rounds [file sj-zip-1-mde-10.1177_23821205261449384.zip › F. GREET Reporting Guideline List.pdf]

GREET 2015 checklist* based upon the TIDieR guidance

BRIEF NAME	Page Location
1. INTERVENTION: Provide a brief description of the educational intervention for <u>all</u> groups involved [e.g. control and comparator(s)].	Pgs 4-5
WHY - this educational process	
2. THEORY: Describe the educational theory (ies), concept or approach used in the intervention.	Pg 4
3. LEARNING OBJECTIVES: Describe the learning objectives for <u>all</u> groups involved in the educational intervention.	Pg 5
4. EBP CONTENT: List the foundation steps of EBP (ask,acquire, appraise, apply, assess) included in the educational intervention.	N/A
WHAT	
5. MATERIALS: Describe the specific educational materials used in the educational intervention.<br>Include materials provided to the learners and those used in the training of educational intervention providers.	Pgs 5-6; Supplement A, B, & C
6. EDUCATIONAL STRATEGIES: Describe the teaching / learning strategies (e.g. tutorials, lectures, online modules) used in the educational intervention.	Pgs 6-7
7. INCENTIVES: Describe any incentives or reimbursements provided to the learners.	N/A
WHO PROVIDED	
8. INSTRUCTORS: For each instructor(s) involved in the educational intervention describe their professional discipline, teaching experience / expertise. Include any specific training related to the educational intervention provided for the instructor(s).	Pg 6
HOW	
9. DELIVERY: Describe the modes of delivery (e.g. face-to-face, internet or independent study package) of the educational intervention. Include whether the intervention was provided individually or in a group and the ratio of learners to instructors.	Pg 5
WHERE	
10. ENVIRONMENT: Describe the relevant physical learning spaces (e.g. conference, university lecture theatre, hospital ward, community) where the teaching / learning occurred.	Pg 5
WHEN and HOW MUCH	
11. SCHEDULE: Describe the scheduling of the educational intervention including the number of sessions, their frequency, timing and duration.	Pg 6
12. Describe the amount of time learners spent in face to face contact with instructors and any designated time spent in self-directed learning activities.	Pg 6
PLANNED CHANGES	
13. Did the educational intervention require specific adaptation for the learners? If yes, please describe the adaptations made for the learner(s) or group(s).	N/A
UNPLANNED CHANGES	
14. Was the educational intervention modified **<u>during</u>** the course of the study? If yes, describe the changes (what, why, when, and how).	N/A
HOW WELL	
15. ATTENDANCE: Describe the learner attendance, including how this was assessed and by whom. Describe any strategies that were used to facilitate attendance.	Pgs 6 & 8
16. Describe any processes used to determine whether the materials (item 5) and the educational strategies (item 6) used in the educational intervention were delivered as originally planned.	Pg 7
17. Describe the extent to which the number of sessions, their frequency, timing and duration for the educational intervention was delivered as scheduled (item 11).	Pg 6
