# Supplementary figures and images for: Professionalism in Practice: A Novel Approach to Integrating Small Doses of Case-Based Professionalism Education Into Monthly Grand Rounds
Source: J Med Educ Curric Dev. 2026 May 7;13:23821205261449384. doi: 10.1177/23821205261449384 (PMC13167374; doi:10.1177/23821205261449384)

# Professionalism in Practice Framework

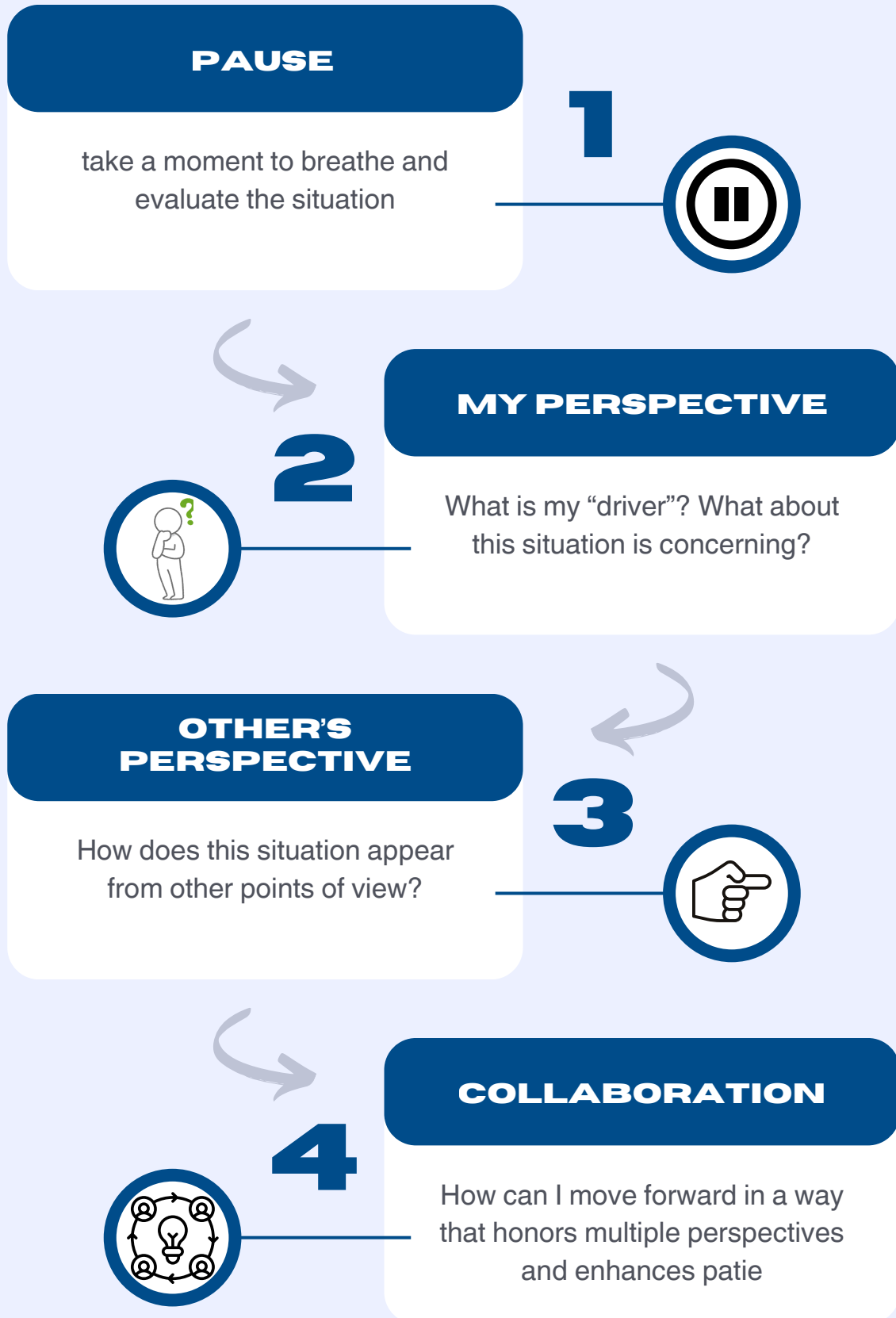

Supplement: Supplemental Material - Professionalism in Practice: A Novel Approach to Integrating Small Doses of Case-Based Professionalism Education Into Monthly Grand Rounds [file sj-zip-1-mde-10.1177_23821205261449384.zip › B. PiP Framework.pdf]
